# Endoscopic Closure of a Pancreatico-Colonic Fistula: A Case Report

**DOI:** 10.7759/cureus.93616

**Published:** 2025-09-30

**Authors:** Ali Al Zaidy, Sameer AlAwadhi, Yousif Alabboudi, Omar Alsayed, Khaled Bamakhrama

**Affiliations:** 1 Gastroenterology, Hepatology, and Endoscopy, Rashid Hospital, Dubai, ARE; 2 General Surgery, Rashid Hospital, Dubai, ARE

**Keywords:** endoscopic approach, gi endoscopy, git endoscopy, minimal invasion, necrotising pancreatitis, pancreatico-colonic fistula, therapeutic colonoscopy

## Abstract

A known complication of severe pancreatitis is the development of localized collections of necrotic tissue, sometimes leading to the formation of abnormal connections (fistulas) with nearby organs. Some fistulas, such as those involving the stomach or small intestine, may allow drainage of fluid naturally and might not require intervention. However, fistulas involving the colon can lead to serious complications ranging from bleeding to sepsis, with less chance of closure, often needing more invasive treatment.

Traditionally, gastrointestinal fistulas were managed surgically, often through bowel resection or diversion procedures. However, these operations carried significant morbidity. More recently, minimally invasive endoscopic techniques, such as the use of clips or tissue sealants, have emerged as effective alternatives and are increasingly reported as successful options for fistula closure.

We describe a case of a 63-year-old female patient with a history of severe pancreatitis complicated by necrotic collections and a fistula involving the pancreas and colon. Using an endoscopic procedure, an over-the-scope clip (OTSC) system (Ovesco Endoscopy AG, Tübingen, Germany) was placed to close the fistula successfully, confirmed by imaging showing no leakage. Following the procedure, her condition improved, she was gradually reintroduced to oral intake, drains were removed, and she recovered without complications.

There are no standardized guidelines for managing these fistulas without surgery or the optimal endoscopic method for closure. A team-based approach involving multiple specialties is essential to provide the safest and least invasive care.

## Introduction

Acute pancreatitis is an acute inflammatory process of the pancreas that may extend to the peripancreatic tissues and involve distant organs. The clinical course can range from a mild, self-limiting illness to severe disease with life-threatening complications. Complications are broadly classified as local or systemic [1]. Local complications include both non-infective forms, such as acute peripancreatic fluid collections, pancreatic pseudocysts, walled-off necrosis, and vascular complications, and infective forms, most notably infected pancreatic necrosis or abscess [1]. The reported incidence of local complications varies, with pseudocysts occurring in up to 10% to 20% of cases and infected necrosis in approximately 5% to 10%. Systemic complications arise from the inflammatory cascade and may manifest as organ failure involving the respiratory, renal, or cardiovascular systems. In addition, disruption of the pancreatic duct and fistula formation, including colonic fistulas, represents a less common but clinically significant sequelae. Fistula formation is a known complication of acute and chronic pancreatitis [1].

A pancreatico-colonic fistula (PCF) is an uncommon but serious complication of acute pancreatitis, frequently arising in the setting of walled-off pancreatic necrosis or infected pancreatic abscess. Pseudocysts may also erode into adjacent colonic segments and give rise to fistula formation [2].

Gastric or small bowel fistulas do not usually warrant intervention, as they spontaneously drain fluid collections. On the other hand, PCFs are usually associated with hemorrhage, sepsis, and persistent infection, which requires more definitive management [3]. 

Among the internal fistulas complicating pancreatitis, PCFs are reported to be the most frequent, whereas pancreatico-gastric fistulas represent the rarest form [4,5]. PCF incidents are reported in 0.4% to 5% of patients with acute pancreatitis [2,4].

Application of conventional endoclips has been reported but not widely used with satisfactory clinical outcomes in some cases [1,3,5-8]. It has been reported that the use of fibrin glue or clips for endoscopic closure of PCFs results in good outcomes. Use of over-the-scope clips (OTSC) for PCF closure has rarely been reported and needs more evaluation [1,3,6,9,10]. Currently, the standard treatment for colonic fistula is open surgical repair [10]. 

## Case presentation

**Figure 1:**
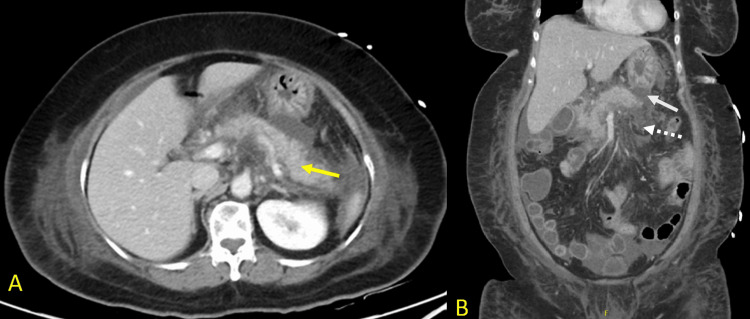
CT scan of the abdomen with contrast A: The axial section shows a diffusely enlarged pancreas (yellow arrow) that appears edematous with significant acute peripancreatic free fluid noted, measuring up to 30 mm in thickness. No evidence of parenchymal necrosis is seen. B: Coronal section, which shows the fluid extending superiorly into the lesser omentum (white arrow) and inferiorly along the inferior transverse mesocolon (dotted white arrow).

A 63-year-old woman presented to the emergency department at Rashid Hospital, Dubai, United Arab Emirates, with a same-day history of epigastric abdominal pain associated with vomiting. She was diagnosed with acute biliary pancreatitis and was admitted for inpatient management, where she was started on intravenous fluid therapy. On the third day of admission, she developed respiratory distress and tachycardia, necessitating transfer to the intensive care unit, where she required noninvasive ventilation. She underwent a CT scan of the abdomen with contrast (Figure 1).

Her condition gradually improved, and she was transferred to the general ward after five days. With continued medical management, she made steady progress and was discharged home 18 days after admission in stable condition.

**Figure 2:**
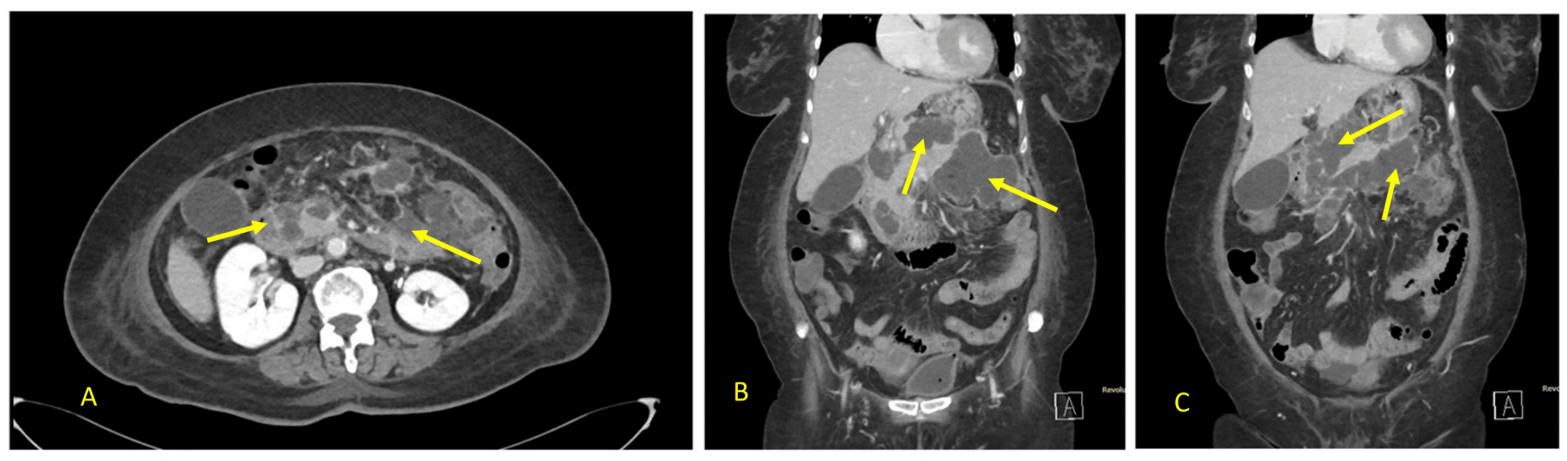
Sequelae of acute pancreatitis with interval progression of peripancreatic fluid A: Axial section of the abdomen with contrast that shows peripancreatic fluid (yellow arrows); B and C: Coronal section of the abdomen with contrast showing walled-off collection encasing the pancreatic tissue (yellow arrows).

Approximately one week later, she presented to the emergency department again with a one-day history of abdominal pain, subjective fever, and associated diarrhea. On examination, she was tachycardic with epigastric tenderness. Laboratory investigations were notable for elevated inflammatory markers (Table 1). She was readmitted for further evaluation and treatment, and intravenous fluids along with supportive care were initiated. She underwent a repeat CT scan of the abdomen with contrast (Figure 2).

**Table 1:** The patient's blood test results H: high; L: low

Component	Result	Reference Range
WBC count	16.1 (H)	3.6 - 11.0 10^3/uL
Hemoglobin	9.7 (L)	12.0 - 15.0 g/dL
Platelet count	278	150 - 410 10^3/uL
C-reactive protein	296.3 (H)	<5.0 mg/L

Antibiotics were started, and the gastroenterology team was consulted. Endoscopic ultrasound (EUS) revealed a large volume of inflammatory and necrotic tissue between the pancreas and the gastric wall, with only a minimal fluid component. The small, non-communicating pockets were not suitable for drainage at that time.

**Figure 3:**
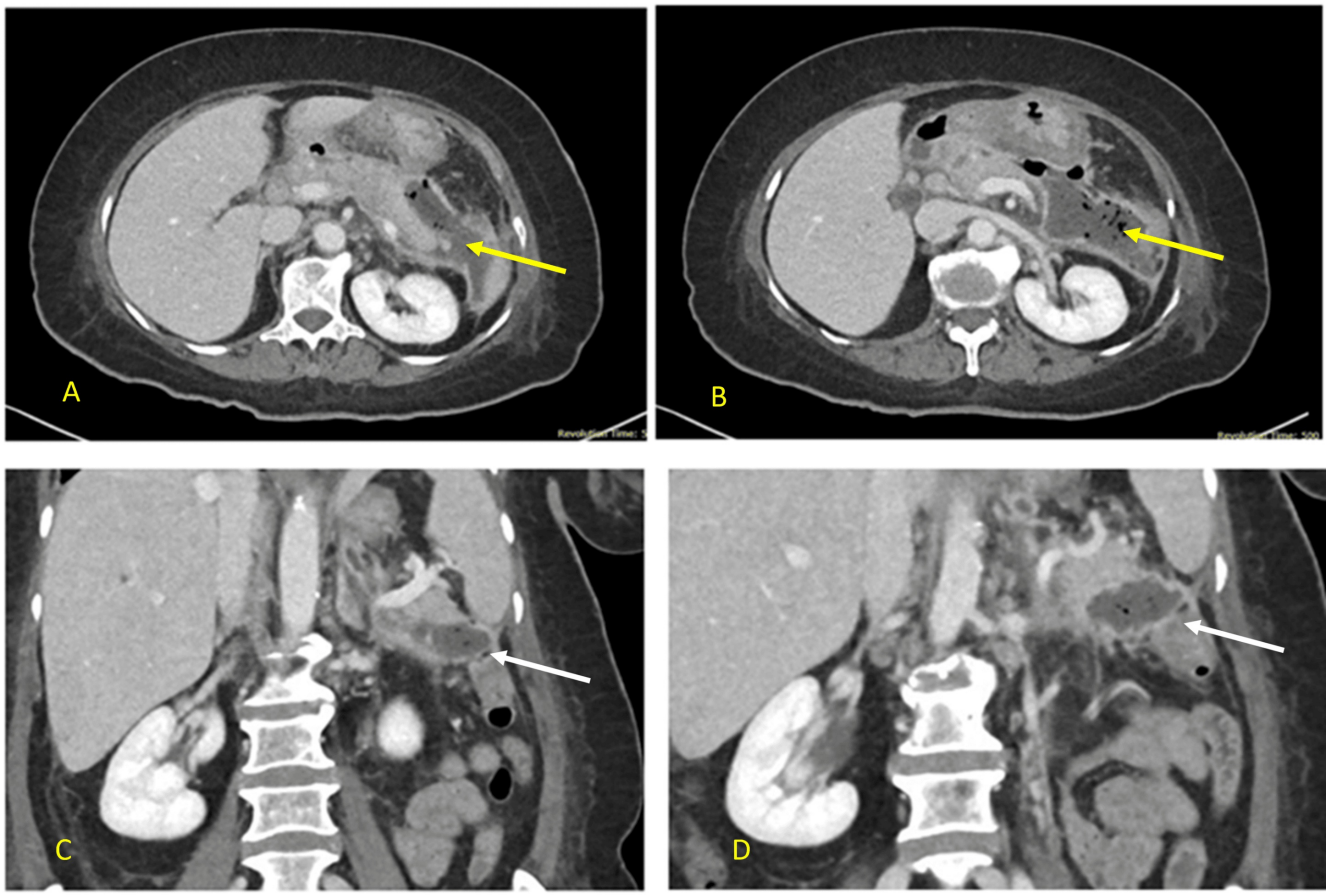
CT scan of the abdomen showing a pancreatco-colonic fistula A and B: The axial section shows a persistent collection with multiple air pockets noted in the tail of the pancreas, with its limbs extending towards the neck (yellow arrow). C and D: a coronal view showing a peripancreatic collection communicating with the splenic flexure, indicative of a fistula (white arrow).

She later underwent image-guided percutaneous drainage with catheter insertion by the interventional radiology team. Her general condition improved, though she continued to report diarrhea. A repeat contrast-enhanced CT scan (Figure 3) demonstrated a persistent collection with multiple air pockets in the pancreatic tail, extending toward the neck and communicating laterally with the splenic flexure, suggestive of a PCF. There was also significant regression of the previously seen collections in the head and neck regions of the pancreas.

**Figure 4:**
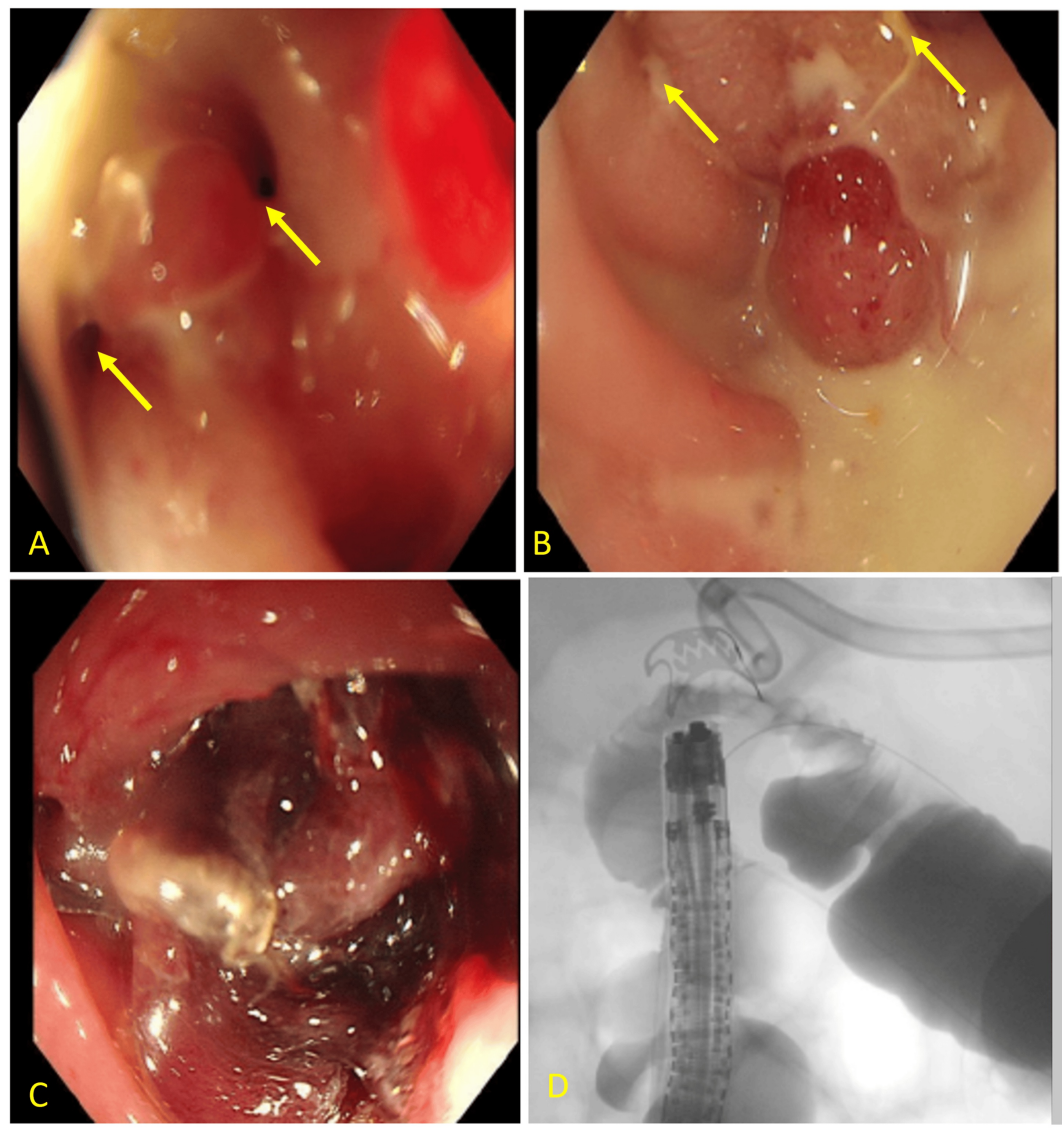
Colonoscopy shows fistulae at the splenic flexure with post clipping and contrast injection A and B: Two fistulae (yellow arrows), both separated by 3 mm from each other in the descending colon 35 cm from the anal verge (splenic flexure). One of the fistulae was 4 mm, and the other was 2 mm. C: After the placement of the over-the-scope clipping. D: A fluoroscopic image with dye injection shows the clip and no contrast extravasation.

A multidisciplinary team comprising general surgery, gastroenterology, and radiology reviewed the case and determined the optimal management strategy. A colonoscopy was performed, with the scope advanced to the terminal ileum, revealing two fistulous openings at the splenic flexure. The fistulae were confirmed by fluorescent imaging, and the patient underwent OTSC (Ovesco Endoscopy AG, Tübingen, Germany) placement, with a single clip successfully applied to both openings. Closure of the fistulae was confirmed using contrast injection and fluorescent imaging (Figure 4).

**Figure 5:**
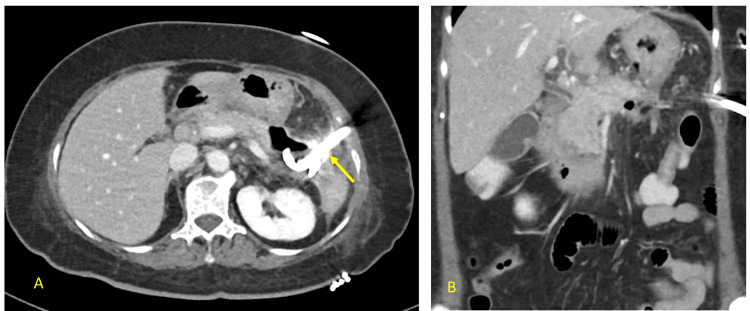
CT scan of the abdomen with contrast shows metallic clip and almost complete resolution of the collection A: Axial section shows the metallic clip (yellow arrow). B: Coronal section shows almost complete resolution of the previously seen collection.

Following the intervention, her condition continued to improve. Inflammatory markers trended downward, the drain output decreased, and the catheter was eventually removed. The patient remained afebrile, pain-free, and able to tolerate oral intake. A post-intervention CT scan showed resolution of the collection (Figure 5).

She was discharged home in good general condition. At a follow-up visit three weeks later, she reported no complaints and had returned to her usual daily activities. She was advised to undergo an elective laparoscopic cholecystectomy.

A follow-up CT scan performed one month after discharge showed near-complete resolution of peripancreatic collection, with only minimal residual fluid at the pancreatic tail and marked reduction in peripancreatic inflammatory changes.

## Discussion

Fistula formation is a recognized complication of both acute and chronic pancreatitis, with PCFs representing a particularly rare entity [1,4,5]. PCFs usually arise from pancreatic necrosis, peripancreatic abscess, or persistent inflammatory processes eroding into the adjacent colon. This pathophysiology explains the severe sequelae associated with PCFs, including persistent infection, sepsis, gastrointestinal bleeding, and high morbidity and mortality, often independent of the underlying severity of pancreatitis [10,11].

Traditionally, surgical management, such as diverting ileostomy with colonic preservation or segmental colonic resection, has been considered the standard of care. However, surgery carries considerable risk, with reported mortality rates reaching up to 40% and complications including multi-organ failure, septic shock, and massive intra-abdominal hemorrhage [10,11].

Historically, the standard management of PCFs was surgical, including resection of the involved colonic segment or diverting ileostomy. While these approaches can be curative, they are associated with significant morbidity and mortality, reported to be as high as 30% to 40% in earlier series. Gao et al. (2020) [11] further demonstrated that surgical intervention in patients with infected pancreatic necrosis and PCFs carried substantial risks, particularly in critically ill patients with multiple organ dysfunction [11].

With the evolution of minimally invasive techniques, endoscopic therapy has emerged as an alternative. Several case reports describe successful PCF closure using endoscopic methods such as fibrin glue, conventional clips, or OTSCs. Von Renteln et al. (2010) [8] and Ito et al. (2013) [10] reported effective use of OTSC in gastrointestinal fistulas, including PCFs, though the number of published cases remains small. More recently, Baliss et al. (2024) [2] described closure of a persistent PCF with OTSC in the context of infected necrosis, highlighting both feasibility and safety. Compared to surgery, endoscopic management demonstrates lower complication rates (approximately 19% vs. 40%) and avoids the morbidity associated with bowel resection and stoma creation [11].

In the present case, the patient was clinically stable, and the fistulous openings were both discrete and endoscopically accessible. Colonoscopy identified two fistulous tracts at the splenic flexure, which were confirmed by fluorescent imaging. An OTSC was deployed, with a single clip effectively sealing both openings. Follow-up demonstrated complete resolution of the fistula and resolution of diarrhea.

The success of OTSC closure in this case can be attributed to several favorable factors: the small, well-defined nature of the fistulous openings; the absence of extensive necrosis or ongoing infection; and the immediate confirmation of closure with both contrast injection and fluorescent imaging. Importantly, this case highlights a unique technical achievement, the successful closure of two distinct colonic fistula openings with a single OTSC device. To our knowledge, this is the first reported instance of such an approach in the context of PCFs. This detail underscores the versatility of the OTSC system in managing anatomically complex fistulas while minimizing the need for multiple devices or procedures.

Although endoscopic closure of PCFs using OTSC has been rarely reported, outcomes appear highly dependent on anatomic accessibility and operator expertise. When these conditions are met, OTSC offers a safe, minimally invasive alternative to surgery, with the potential to reduce morbidity, shorten recovery, and avoid the risks associated with major surgical intervention. Furthermore, the technical efficiency demonstrated in this case suggests that OTSC may have an expanded role in the treatment of complex pancreatitis-related fistulas, particularly when multiple tracts are present.

Nevertheless, certain limitations must be acknowledged. The success in this patient may not be generalizable to larger or more inflamed fistulas or to cases complicated by extensive necrosis and ongoing infection. Additionally, the long-term durability of the closure remains uncertain, as follow-up was limited. Future studies and additional case reports are needed to better define patient selection criteria, procedural success rates, and long-term outcomes of OTSC closure in PCFs.

## Conclusions

This case demonstrates that endoscopic closure of a PCF using the Ovesco OTSC system can be a safe and effective alternative to surgical management in selected patients. Post-discharge follow-up confirmed complete resolution of the fistula.

Given the rarity of this complication and the limited literature on OTSC use for PCFs, management should involve a multidisciplinary team, including general surgery, gastroenterology, radiology, and internal medicine. Such an approach helps optimize outcomes, minimize surgical risks, and support the development of minimally invasive strategies for this challenging condition.
